# Focal Therapy for Prostatic Carcinoma

**DOI:** 10.1155/2012/703923

**Published:** 2012-12-30

**Authors:** Eric Barret

**Affiliations:** Institut Montsouris, Université Paris Descartes, France

Medical therapeutics has solidly evolved in the last decades based on bright ideas and hard work. Thinking outside the box remains essential if one's idea is to make his/her specific fields move forward. Some years ago we were lucky to become part of the surgical evolution in radical prostatectomy; the development of laparoscopic radical prostatectomy followed by the robotic version of the procedure not only transformed the actual operation, but also brought up a minutely precise analysis of surgical outcomes. Prostate cancer remains one of the most rapidly evolving areas in medicine and perhaps after a massive application of surgical concepts to treat disease, we have come to the point to question ourselves about *overtreatment. *This is how the genius idea of active surveillance was born and the evolution of this approach has offered thousands of patients the logical option of a less aggressive approach to deal with a positive prostatic biopsy. 

More recently, many of us have questioned both active surveillance and any other more aggressive treatment options, based on an oncological concept previously applied with success in other organs: partial treatment (partial mastectomy, partial nephrectomy, and partial cystectomy), meaning to treat only the diseased portion of an organ and not the whole thing.

Today, this is how we aim to make the field move forward in prostate carcinoma therapeutics; with focal therapy, a logical intermediate option to treat patients only was the positive biopsies are located aiming to obtain solid cancer control while maintaining quality of life.

We were pleased to guest this special issue, as we consider there is much to do in order to further improve this novel idea, that, as any other idea ever presented in medicine, remains a rather controversial issue where once again brilliant thoughts must be backed up with enormous effort to rapidly evolve beyond the proof of concept.

Focal therapy for prostate cancer should be established on three basic concepts: first, a reliable determination of cancer location within the prostate, second, a convenient type of energy providing safe and reliable intervention on the diseased portions of the organ, and third, a follow-up approach allowing physicians to objectively decide on further actions to control the disease. The mentioned concepts have not yet been completely defined and a great part of today's research is focused on the improvement of these elements.

We would like to highlight some of the manuscripts presented in the present special issue. To set up the ground for the subject, Al B. Barqawi etal. provide us with a rather solid review of epidemiological and screening studies, prostate cancer statistics, and information on patient outcomes. The manuscript presents the actual situation on prostate cancer therapeutics and how focal therapy will eventually define its role. 

 T. Nomura and H. Mimata present a summary of the recent data regarding focal therapy for prostate cancer and different energies have been deployed in this field. Moreover, they state today's limitations and the expectations we have for the future regarding the evolution of the technique.

Regarding available energies for Focal therapy, U. Maestroni et al. brought the analysis of their complications with high intensity focused ultrasound; 89 patients were analysed to confirm the oncological effectiveness of the procedure and the most related undesired events they observed in their experience. 

P. Colin et al. performed a review on the role of laser energy in Focal Therapy and how the application of laser has been evolved from phase I assays until the future design of solid trials to objectively determine the oncologic efficacy in the long term.

In a rather interesting manuscript, A. K. Jain and R. D. Ennis introduce the approach of differential therapy where the entire prostate is treated to a lower intensity and the tumor areas to high intensity. They review the available experience in the subject with external beam radiation, high-dose rate brachytherapy, and low-dose rate brachytherapy. 

Finally, M. Morita and T. Matsuura present a very provocative paper featuring the application of old, good, and reliable transurethral resection of the prostate cancer treatment, with a subset of their population approached with a focal resection and featuring stable PSA between 0.007 and 0.4 ng/mL up to two-year followup and with a low complications rate. 

Starting from the available knowledge and technology, Focal therapy is continuously developing from its infancy and a more mature era is awaiting. Technological development will be essential in the evolution of this approach; from imaging to tissue treatment, we must work together with technological scientists to bring to the patients side the tools on which we will base highly precise diagnosis, effective tissue treatment, and reliable oncological followup.

*Eric Barret*

